# Editorial: Lupus and the Brain: Advances in Neuropsychiatric Systemic Lupus Erythematosus

**DOI:** 10.3389/fmed.2019.00052

**Published:** 2019-03-26

**Authors:** Antonis Fanouriakis, George Bertsias, Marcello Govoni

**Affiliations:** ^1^Rheumatology and Clinical Immunology Unit, “Attikon” University Hospital, Athens, Greece; ^2^Rheumatology, Clinical Immunology and Allergy, University Hospital of Heraklion, Heraklion, Greece; ^3^Section of Rheumatology, Department of Medical Sciences, University of Ferrara, Azienda Ospedaliero-Universitaria Sant'Anna Ferrara, Ferrara, Italy

**Keywords:** systemic lupus erythematosus, central nervous system, attribution, diagnosis, biomarkers

As systemic lupus erythematosus (SLE) is a notoriously demanding disease, involvement of the nervous system, collectively termed neuropsychiatric SLE (NPSLE), represents the foremost diagnostic and therapeutic challenge for the treating physician. Reasons for this include, but are not limited to, the wide heterogeneity of its presentation, the lack of specific markers and a diagnostic “gold-standard,” as well as the paucity of well-designed clinical trials to guide therapy (1). In this Research Topic for the Frontiers in Medicine, four articles by distinguished contributors touch upon different aspects of NPSLE, in an attempt to elucidate the optimal approach to this challenging group of patients.

In 1999, the American College of Rheumatology (ACR) research committee published a set of case definitions for 19 NPSLE syndromes, in an effort to homogenize terminology for research and clinical practice purposes. These case definitions involve both the central and the peripheral nervous system, are categorized into focal and diffuse and have a wide heterogeneity that ranges from overt manifestations such as stroke, seizures and psychosis, to headache or subtle abnormalities of cognitive function (2). An “outsider” of the NPSLE field would rightfully wonder straight away: How can a patient with headache, a patient with psychosis and a patient with peripheral neuropathy, three very different conditions, be grouped under the same “umbrella” term (which implies common pathophysiology and possibly treatment)? Are these patients the same, or should they be approached in the same way?

The now considered “classic” population-based study by Ainiala et al., published shortly after the ACR NPSLE definitions, questioned the value of including the so-called “common” or “minor” manifestations (including headache, anxiety disorder, mild mood disorder and cognitive impairment, and polyneuropathy without electrophysiologic confirmation) in the spectrum of NPSLE (3). By excluding these manifestations, specificity for the diagnosis of “frank” NPSLE increased from 46 to 93%, since the former are very common also in the general population. This, and other observations, have steered the discussions as to what actually constitutes “NPSLE.” The actual existence of “lupus headache,” as a distinct NPSLE syndrome, has been questioned by some authors (4, 5). Bearing these in mind, in this Research Topic, Vivaldo et al. critically revisit the original ACR nomenclature to comment on its advantages, but also potential pitfalls. While the authors acknowledge the major step forward accomplished by the ACR nomenclature to homogenize NPSLE research, they suggest that a more comprehensive model, taking into account also the issue of attribution, could move the field forward, to optimize patient care.

The issue of attribution represents an important unmet need in NPSLE (6). In a lupus patient with a given neuropsychiatric manifestation, correctly attributing the latter to the disease *per se* or not has important therapeutic implications. Given that in NPSLE “nothing is specific,” various attempts have been made to construct sets of rules and attribution models. In a relevant review, Bortoluzzi et al. present an overview of previously proposed “hints” for attribution, focusing on their own formidable effort, which led to the attribution algorithm of the Italian Study Group for NPSLE (7). The novelty of the Italian model was the inclusion of “favoring” factors, i.e., factors whose presence is supportive of diagnosing primary NPSLE. These include the presence of generalized disease activity, abnormal brain imaging and cerebrospinal fluid (CSF) analysis, aPL positivity and others. This algorithm has now been independently validated and may provide significant help to physicians and center with less expertise in SLE; it also offers, to researchers in the field, a valuable tool for a more rigorous and standardized selection of NPSLE patients for epidemiological studies and clinical trials (8). Nevertheless, to emphasize the indispensable role of multidisciplinary and longitudinal judgment in NPSLE, a subsequent study testing the proposed models found that, upon re-evaluation after 3–8 months from initial attribution, almost 15% of patients initially classified as primary NPSLE were deemed as having neuropsychiatric events not attributed to SLE (9).

Irrespective of the clinical syndrome, the pathophysiology of NPSLE is traditionally considered a combination of autoantibody-mediated neuronal or vascular injury, intrathecal production of inflammatory cytokines, disruption of the blood-brain barrier, and accelerated atherosclerosis (1). A certain degree of cerebral vasculopathy is evident in NPSLE by the common finding of white matter T2-hyperintense lesions in conventional brain MRI of patients with NPSLE, but also from multiple studies using novel brain imaging techniques, which show hypoperfusion even in normal appearing white matter (10–13). Teixeira and Tam have performed a systematic literature review, to provide modern insights on the pathophysiology of premature and accelerated atherosclerosis in SLE. Importantly, the authors provide a thorough overview of molecular biomarkers that have been tested in relation to cardiovascular/cerebrovascular events and mortality; they also outline the various imaging surrogate markers for atherosclerosis, including flow-mediated dilatation of the brachial artery, carotid intima media thickness and pulse wave velocity. Albeit these studies have added valuable information, no single surrogate marker (molecular or imaging) has yet shown a clear association with “hard” endpoints, i.e., vascular events or cardiovascular mortality.

Along the same lines, in a Perspective article, Magro-Checa et al. present the current state-of-the-art regarding the utility of biomarkers (laboratory and neuroimaging) for prediction and diagnosis of NPSLE, in particular. The authors give a comprehensive review of serum and CSF biomarkers; notably, from the bulk of experimental biomarkers, only serum lupus anticoagulant and CSF interleukin-6 have reached clinical usefulness. Following also an extensive description of the various novel brain imaging techniques which have been utilized in NPSLE, Magro-Checa et al. end with attractive proposals to improve the scant performance of the pursuit for biomarkers in NPSLE, ranging from definition of NPSLE and better characterization of patient groups, to the combination of laboratory and multimodal neuroimaging markers.

In conclusion, our understanding of NPSLE has witnessed significant advances over the last decades. Nevertheless, to optimize patient care, future research should focus on a better characterization of patient cohorts, through multicentre international collaborations, as well as establishment of universally accepted guidelines for diagnosis, evaluation of disease activity, identification of more specific outcome measures and assessment of response to treatment.

## Author Contributions

All authors listed have made a substantial, direct and intellectual contribution to the work, and approved it for publication.

### Conflict of Interest Statement

The authors declare that the research was conducted in the absence of any commercial or financial relationships that could be construed as a potential conflict of interest.
